# Virtually delivered family‐based eating disorder treatment using an enhanced multidisciplinary care team: A case study

**DOI:** 10.1002/ccr3.4173

**Published:** 2021-05-06

**Authors:** Megan Hellner, Cara Bohon, Samuel Kolander, Erin Parks

**Affiliations:** ^1^ Equip Health San Diego CA USA; ^2^ Stanford University School of Medicine Stanford CA USA; ^3^ New York State Psychiatric Institute New York NY USA

**Keywords:** adolescents, anorexia nervosa, family therapy, feeding and eating disorders, mentors, telemedicine

## Abstract

Both patients experienced meaningful clinical improvements with this virtual approach and the augmented treatment team in regards to weight gain, acceptability, and clinical assessment scores. These findings offer preliminary support for this model.

## Graphical abstract

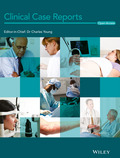

## 1 INTRODUCTION

While family‐based treatment (FBT) is the recommended treatment for adolescents and young adults with eating disorders, it is often not geographically accessible, and outcomes can be negatively impacted by misalignment between treatment providers. The aim of this case report is to describe outcomes of two patients treated virtually with an augmented team approach to provide preliminary evidence of feasibility and efficacy. Two patients were enrolled at Equip for 4 weeks of treatment. Outcome measures included weight, satisfaction ratings, engagements, and survey responses to the Generalized Anxiety Disorder Scale, Patient Health Questionnaire, Eating Disorder Examination Questionnaire. Sessions were held via telehealth with four providers (therapist, dietitian, peer mentor, and family mentor). Patient one gained 6.4 lbs/2.9 kg, and patient two gained 4.2 lbs/1.9 kg during the trial. Eating disorder symptom scores also decreased for each patient. Patients attended 18 and 16 total sessions, respectively, and both carers and patients reported that they would “definitely recommend” the treatment to a friend or family, suggesting strong acceptability. Weight gain continued for both patients at 4‐week follow‐up (2 lbs/0.9 kg and 4.8 lbs/2.2 kg, respectively). These findings offer preliminary support for this treatment resulting in meaningful clinical improvements. Future research should examine this treatment in larger samples, with longer follow‐up periods and comparison treatments.

Eating Disorders are life‐threatening conditions characterized by a marked disturbance in how one experiences weight (DSM‐V). Further, eating disorders have the second highest standardized mortality rates of all psychiatric illnesses.^1^ Family‐based treatment (FBT) is well established as the recommended treatment for anorexia nervosa (AN).^2^ Delivery of FBT requires use of a specialized provider trained in the approach, which presents a challenge for families residing in “treatment deserts” without access to specialized services.

Despite the abundance of evidence supporting FBT, a first‐line treatment, an evaluation of therapeutic approaches used by community providers treating eating disorders demonstrated that the majority of providers do not adhere to best practice standards/evidence‐based practices. The approach most commonly used by psychotherapy providers is “eclectic” (43%), and addiction‐based/“12‐step” approaches (26%).^3^ Further, the majority (57%) received no clinical training or supervision on eating disorders. The most common reasons for choice of approach are compatibility with clinicians' style and training, and “appropriateness” based on clinical judgment. Only a minority endorsed choosing an approach because it was supported by evidence, and it is well documented that favoring clinical judgment over well‐established interventions results in poorer outcomes.^3^, ^4^ As such, innovative approaches for improving uptake of FBT for treatment of eating disorders are needed.

Telehealth approaches to delivering care offer an innovative solution and are becoming more widespread, particularly as a result of the COVID‐19 pandemic.^5^ Outcomes for care delivered via telehealth rival those of in‐person care across a number of conditions, while also addressing issues relating to access to specialized care.^6^ Since the onset of the COVID‐19 pandemic, there's been a staggering jump in mental health conditions.^5^ Rates of depression, anxiety, substance use, and suicidality have risen significantly since 2019,^5^ and persons with existing psychiatric issues are particularly vulnerable. Prevalence of comorbid anxiety, depression, and substance use disorders are increased in individuals with eating disorders, and there is an expected increased burden on the healthcare system in the years ahead.^7^, ^8^ The social isolation and challenges with service access highlight a need for increased use of telehealth for delivery of evidence‐based treatment. A recent report described anticipated challenges (such as rapport building, medical, and weight monitoring) and proposed solutions for delivering FBT virtually.^9^ Findings suggest that the merits of virtually delivered FBT outweigh limitations and that adaptations can be developed to minimize many of the challenges.^9^, ^10^

While FBT is regarded as the recommended treatment for anorexia nervosa, and while it can be delivered remote using Telehealth solutions, there is a continued need for testing further improvements to the approach, given that over half of those who undergo FBT do not show remission by the end of the treatment course.^11^ A number of studies have examined benefits and challenges of providing an individual with a “lived experience” mentor, defined as one who has a shared struggle and has since recovered. Mentorship programs have been shown to improve quality of life and decrease psychiatric symptoms in other health conditions.^12^ When looking specifically at mentorship in eating disorders, reported benefits to mentees include feelings of belonging, comfort, hope, connection, and a unique sense of support due to the mentor's lived experience. One study examined the influence of parent to parent support on outcomes of FBT for anorexia nervosa by randomizing 20 families to receive either standard FBT or FBT with the addition of parent consultation. The rate of weight restoration was found to be higher in the group receiving parent to parent support.^13^ More recently, adjunctive peer mentorship has been shown to reduce mentees’ symptoms of anxiety, depression and body dissatisfaction, and to improve engagement in treatment.^14^

We enrolled two families in a beta trial from April to May 2020, providing 4 weeks of no‐cost treatment. The conventional treatment team consisting of a family therapist and registered dietitian was enhanced by the addition of a “lived experience” arm consisting of a peer mentor and family mentor. The care team held individual sessions weekly (at minimum, with additional sessions available upon request) with carers, patient, or both, via Equip's HIPAA compliant telehealth platform. For these trial cases, medical care was provided locally for participants outside of the Equip system. We tracked outcomes and solicited feedback via a combination of surveys and assessments, typically at both baseline and discharge. The primary outcome of this study was weight change in participants with the expectation that each patient gain an average of one pound per week during the 4 weeks of treatment. Secondary outcomes are changes in scores on the Generalized Anxiety Disorder Scale (GAD‐7), Patient Health Questionnaire (PHQ‐9), and Eating Disorder Examination Questionnaire (EDE‐QS) scores over time, with the expectation that scores on these scales would decrease during the 4 weeks of treatment. In addition to these clinical measures, we aimed to evaluate comfort level with the technological aspects of the telehealth platform.

In summary, family‐based treatment is regarded as “best practice and first‐line treatment” for eating disorders. The use of mentorship support from people with lived experience has also been documented to be an effective model for enhancing treatment outcomes. To our knowledge, the combination of FBT and peer support has not been utilized to deliver eating disorder treatment. Our objective was to share preliminary outcomes for the two case study patients treated using this augment virtual FBT approach.

## 2 METHODS

### 2.1 Overview of study design

Equip Behavioral Health enrolled two families in a beta trial from April to May 2020, providing 4 weeks of no‐cost treatment. The conventional treatment team consisting of a family therapist and registered dietitian was enhanced by the addition of a “lived experience” arm consisting of a peer mentor and family mentor. The care team held individual sessions with carers, patient, or both, via Equip's HIPAA compliant telehealth platform. For these trial cases, medical care was provided locally for participants outside of the Equip system. We tracked outcomes and solicited feedback via a combination of surveys and assessments, typically at both baseline and discharge. The primary outcome of this study was weight change in participants with the expectation that each patient gain an average of one pound per week during the 4 weeks of treatment. Secondary outcomes are changes in mood and eating disorder behaviors over time, with the expectation that scores of these measures would decrease during the 4 weeks of treatment. In addition to these clinical measures, we aimed to evaluate comfort level with the technological aspects of the telehealth platform.

### 2.2 Participant recruitment and eligibility

Two patients with a diagnosed eating disorder (participant 1 with anorexia nervosa and participant 2 with atypical anorexia) participated in the beta trial. Patients were recruited by Equip providers through postings made on social media‐based professional networking groups and listservs. Assessments were performed by a licensed doctoral level psychologist. Inclusion criteria required that the patient live at home with carers, that carers were willing and able to engage fully in treatment (including attending family therapy, dietary sessions, family mentor sessions, and carer skills groups) and that carers had the ability to supervise eating, if needed. Patients were excluded from participation if they were medically unstable or struggled with any of the following: actively suicidality or past suicide attempts, borderline personality disorder or other personality disorder, significant substance above, or significant current nonsuicidal self‐injury. Patients were also excluded if carers had an active eating disorder, a significant personality disorder, engaged in neglectful or abusive behavior toward the patient, or were unwilling/unable to prioritize the patient's needs. These criteria are representative of common indications and contraindications used outside of research settings when clinicians are using clinical judgment to determine whether or not a family/patient will benefit from family‐based treatment. This trial met criteria for “chart review”, and thus Equip received approval for exemption status from Western Institutional Review Board.

### 2.3 Treatment approach

Each treatment team contained a family therapist, dietitian, peer mentor, and family mentor. Each patient and/or carer for the patient met with each of the providers weekly or more, depending on need. As such, patients and carers had a minimum of four sessions each week. Unlimited messages via a chat box were also available in the Telehealth platform between patients and providers and within the provider team. Patients were asked to weigh twice a week and submit their weigh in the platform. Weights were graphed and available for viewing by providers. The therapist worked with the patient and carers utilizing an FBT approach, which aims to empower parents to take charge of renourishing their child. Parents are tasked with making decisions around how much, when, and what the child is eating with the support of a specialized team.^11^ The therapist also incorporated CBT and DBT techniques where appropriate to challenge rigidities in cognitions and maladaptive coping skills. The dietitian used the patient's current weight and growth charts to create an informed target weight range for the patient. The dietitian additionally prescribed a meal plan and exercise plan to safely and effectively allow for appropriate weight restoration. The peer mentor engaged in weekly meetings with the patient, offering support and judicious self‐disclosure, and acting as a role model for recovery. The family mentor met with the patient's carers to offer support and advice for ways to provide effective nourishment and limit unhealthy behaviors. The Equip treatment team collaborated with an in‐person medical provider to monitor patient's medical stability including vital signs and laboratory tests.

### 2.4 Measures

#### 2.4.1 Generalized anxiety disorder scale

The Generalized Anxiety Disorder Scale (GAD‐7) is a widely used, validated and reliable self‐report tool for screening, diagnosing, and assessing severity of an anxiety disorder. Individuals are asked to rate seven items pertaining to anxiety symptoms which are scored from 0 to 3, and the score is totaled. Anxiety is rated as either minimal, mild, moderate, or severe. This scale was administered at baseline, and again upon discharge.^15^

#### 2.4.2 Patient health questionnaire

The Patient Health Questionnaire (PHQ‐9) is a brief, well‐validated, and reliable diagnostic tool for depression. Individuals are asked to rate nine items pertaining to depression symptoms they have found bothersome over the last 2 weeks from “0” (not at all) to “3” (nearly every day). Depressive symptoms are then rated as minimal, mild, moderate, moderately severe, or severe. This scale was administered at baseline, and again upon discharge.^16^

#### 2.4.3 Eating disorder examination questionnaire short form

Patients completed the Eating Disorder Examination Questionnaire Short Form (EDE‐QS) at baseline, and again at discharge. This scale is an abbreviated 12‐item version of the 28‐item Eating Disorder Examination Questionnaire (EDE‐Q) survey. It is a well‐validated and reliable measure which assesses eating disorder symptom severity. Scores range from 0 to 36, with higher scores suggesting increasing severity of eating disorder behavior.^17^

#### 2.4.4 Satisfaction rating

Upon completion of the beta trial, carers and the patient were asked the following question: “On a scale from 0 to 10, how likely are you to recommend Equip to a friend or family?” with zero representing “Would definitely not recommend” and 10 representing “Would definitely recommend.”

#### 2.4.5 Engagement

Engagement with the therapist, dietitian, peer mentor, and family mentor was measured by counting the number of sessions attended by both carers and patient, and number of total messages exchanged within the Equip platform's “chat” feature.

#### 2.4.6 Weight

Carers were instructed on proper procedure in regard to monitoring weight upon onboarding in their first meeting with the Registered Dietitian. They were instructed to check weight twice weekly in minimal clothing, after voiding, and prior to food or beverage consumption. Weight was communicated via text from carers to Equip's HIPAA compliant platform. Carers also provided a weight update at 4 weeks postdischarge. The target weight for each patient was determined using each patient's CDC age adjusted BMI growth chart. Growth chart data are used rather than “ideal body weights” based upon established norms for BMI in an effort to realign the patient with their pre‐eating disorder growth and developmental trajectories.

## 3 RESULTS

Patient characteristics and outcome data are reported in Table 1. Two patients with a diagnosed eating disorder (participant 1 with anorexia nervosa and participant 2 with atypical anorexia) participated in the beta trial. In terms of our primary outcome, both patients gained at least 1 pound (lb)/0.5 kg per week over the course of the trial (6.4 lbs/2.9 kg and 4.2 lbs/1.9 kg in 4 weeks respectively). For secondary outcomes, scores for Eating Disorder Examination Questionnaire decreased by 7 and 12 points, suggesting a marked decrease in eating disorder symptoms. There are no significant changes in Patient Health Questionnaire and Generalized Anxiety Disorder Scale scores, with one participant scoring “mild” at baseline and discharge on both assessments, and the other scoring “moderate” at baseline and discharge on both assessments. Patients and carers attended 18 and 16 sessions, respectively, though carers of patient 2 engaged with the team via the “chat” feature at a much higher frequency. Both carers and patients reported that they would “definitely recommend” Equip to a friend or family.

**TABLE 1:** Patient characteristics and trial data (baseline and discharge)

	Patient 1	Patient 2
Diagnosis	Anorexia nervosa	Atypical anorexia nervosa
Age	20	15
# Sessions attended	18	16
Session breakdown		
Therapist	5	4
Registered dietitian	4	5
Family mentor	5	3
Peer mentor	4	4
# Messages exchanged	19	43
EDE‐QS (Baseline, discharge, change)	11, 4, −7	24, 12, −12
PHQ‐9 (Baseline, discharge, change)	4, 5, +1	8, 7, −1
GAD‐7 (Baseline, discharge, change)	2, 1, −1	6, 6, ±0
Height, inches/cm	67/170	59/150
Weight, lbs/kg (Baseline, discharge, change)	111.6/50.6, 118.0/53.5, +6.4/2.9	97.0/44.0, 101.2/45.9, +4.2/1.9
Weight, percent of target weight (baseline, discharge)	82.7, 87.4	92.4, 96.4
Weight, lbs/kg, 4 wk post d/c	120/54.4	106/48.1
Target weight, lbs/kg	135/61.2	105/47.6
Satisfaction score (patient)	9	8
Satisfaction score (parent)	10	10

## 4 DISCUSSION

The beta trial patients demonstrated a positive response to Equip's treatment model, as both patients gained at least one pound per week during the trial. In terms of secondary outcomes, there was a decrease in Eating Disorder Examination Questionnaire scores for both patients however Patient Health Questionnaire and Generalized Anxiety Disorder Scales remained stable and subclinical over the course of treatment. Further, both patients continued to progress with weight restoration after treatment ended. Patients and carers were both highly engaged during the trial with both traditional providers (therapist and dietitian) as well as mentors. This was promising given potential for loss of rapport or connection when care is virtually delivered. Having the additional support from the “lived experience” vantage point with the addition of mentors to the treatment team may overcome perceived limitations related to virtual care. Carers and patients were very satisfied with the approach overall, and verbally expressed finding great value in the unique support received from all four team members.

This case study's results are limited by the short intervention period and use of nonvalidated measurement tools in regard to satisfaction and engagement scores. Further, we have limited data postdischarge (weight only). Engagement and satisfaction may be influenced by treatment being at no cost, and thus expectations of paid patients may differ. Also, survey responses were given via self‐report and, thus, may pose a threat to reliability. Additionally, further investigation is needed to ensure that other patients show similar improvements with the treatment approach, although the report of these two cases is promising.

These findings offer preliminary support for achieving meaningful clinical outcomes using Equip's virtually delivered augmented FBT model. If future studies of this approach that include larger samples, longer follow‐up periods, and comparison treatments replicate the outcomes from these cases, the treatment approach could be crucial for addressing limitations in access to care for families living far from trained FBT specialists.

## CONFLICT OF INTEREST

None declared.

## AUTHOR CONTRIBUTIONS

MH: involved in conception of the study, review of literature, data analysis and interpretation, initial draft of the manuscript, critical revision of manuscript, and approval of final version prior to submitting for publication.SK: involved in interpretation of data, initial draft of the manuscript, critical revision of the manuscript, and approval of the final version prior to submitting for publication. CB: involved in review of literature, Interpretation of data, critical revision of the manuscript, and approval of the final version of the manuscript prior to submitting for publication. EP: involved in conception of the study, collection of data, and final approval of the version of the manuscript prior to submitting for publication.

## CONSENT STATEMENT

Published with written consent of the patient.

## Data Availability

The data that support the findings of this study are available from the corresponding author upon reasonable request.
